# Ocular lesions in hereditary hemorrhagic telangiectasia: genetics and clinical characteristics

**DOI:** 10.1186/s13023-020-01433-5

**Published:** 2020-06-29

**Authors:** Inés Gómez-Acebo, Sara Rodríguez Prado, Ángel De La Mora, Roberto Zarrabeitia Puente, Beatriz de la Roza Varela, Trinidad Dierssen-Sotos, Javier Llorca

**Affiliations:** 1 Facultad de Medicina, Universidad de Cantabria, Avda. Herrera Oria s/n, 39011 Santander, Spain; 2 IDIVAL, Santander, Spain; 3 CIBER Epidemiología y Salud Pública (CIBERESP), Madrid, Spain; 4 Department of Ophthalmology, Hospital Sierrallana, Torrelavega, Cantabria Spain; 5 Department of Internal Medicine, Hospital Sierrallana, Torrelavega, Cantabria Spain; 6 Centro de Investigación Biomédica en Red de Enfermedades Raras (CIBERER), Madrid, Spain

**Keywords:** Hereditary hemorrhagic telangiectasia, HHT, Osler-weber-Rendu, ENG, ACVRL1/ALK1

## Abstract

**Background:**

The aim of our study is to study the association between eye lesions in Hereditary Hemorrhagic Telangiectasia (HHT) and other signs of the disease, as well as to characterize its genetics.

**Methods:**

A cross-sectional study was conducted of a cohort of 206 patients studied in the HHT Unit of Hospital de Sierrallana, a reference centre for Spanish patients with HHT. Odds ratios for several symptoms or characteristics of HHT and ocular lesions were estimated using logistic regression adjusting for age and sex.

**Results:**

The ocular involvement was associated with being a carrier of a mutation for the ENG gene, that is, suffering from a type 1 HHT involvement (OR = 2.09; 95% CI [1.17–3.72]). *p* = 0.012). In contrast, patients with ocular lesions have less frequently mutated ACVRL1/ALK1 gene (OR = 0.52; 95% CI [0.30–3.88], *p* = 0.022).

**Conclusions:**

In conclusion, half of the patients with HHT in our study have ocular involvement. These eye lesions are associated with mutations in the ENG gene and ACVRL1/ALK1 gene. Thus, the ENG gene increases the risk of ocular lesions, while being a carrier of the mutated ACVRL1/ALK1 gene decreases said risk.

## Background

Hereditary hemorrhagic telangiectasia (HHT) or Rendu-Osler-Weber syndrome is a rare disease that affects between 5000 to 8000 people worldwide [1], although, given the clinical variability of the disease and subclinical forms, it is believed that the figures are underestimated. The HHT presents a wide geographic variability, the highest prevalence rate being among the Afro-Caribbean population of the islands of Curaçao and Bonaire, with 1 case in 1331 inhabitants due to a probable founder effect [2].

HHT is characterized by the presence of vascular malformations; its diagnosis is based on Curaçao’s clinical criteria: recurrent epistaxis, multiple telangiectases in skin and mucosae, visceral involvement (including, for instance, arteriovenous malformations in lung, brain or liver) and first degree relative with HHT. Three or four criteria need to be fulfilled for the diagnosis to be established [3]. HHT is inherited with an autosomal dominant pattern. Approximately 75% of cases are caused by mutations in the Endoglin (ENG) gene, responsible for the HHT1 subtype, and ACVRL1ACVRL1/ALK1(activin A receptor like kinasa type 1), responsible for the HHT2 subtype. About 1–3% of patients with a clinical diagnosis or 10% of patients with a negative genetic test for ENG and ACVRL1/ALK1are carriers of mutations in the MADH4 (mother against decapentaplegic homolog 4) gene, that codifies for Smad 4 [4, 5]. The HHT Subtype also has geographical preferences, with HHT1 being more frequent in North America [6, 7] and in northern Europe [8], while HHT2 is more prevalent in Mediterranean countries like Italy [9, 10], France [11] and Spain [12].

Although eye involvement is frequent, few studies have described its characteristics and its relationship with other HHT features [13–17], and they were purely descriptive. The main goal of this paper is to study the association between eye lesions in HHT and other signs of the disease, as well as to characterize its genetic background. In order to do it, we carried out a cross-sectional analysis of a cohort of 206 patients studied in the HHT Unit of Hospital de Sierrallana, Spain.

## Methods

### Setting and patients

The study was conducted in the HHT Unit of the Sierrallana Regional Hospital (Torrelavega, Cantabria), Spanish national reference centre for HHT. Patients were included if they have a confirmed diagnosis of HHT according to Curaçao criteria and/or genetic test between March 2003 and September 2013. Patients who do not reside in Spain or without Spanish nationality have been excluded from the analysis. All patients were screened by the Ophthalmology Department to assess the presence of ocular lesions associated with HHT.

All procedures performed in studies involving human participants were in accordance with the ethical standards of the institutional and / or national research committee, and with the 1964 Helsinki Declaration and its later amendments or comparable ethical standards. The specific study reported here was approved by the Ethical Committee of Clinical Research of Cantabria. Informed consent was obtained from all individual participants included in the study.

### Diagnosis procedure (Fig. 1)

Patients accomplishing 3 or 4 Curaçao criteria were diagnosed as HHT. Patients fulfilling only 1 or 2 Curaçao criteria were diagnosed as HHT only if they also carried a mutation in ACVRL1/ALK1, ENG or MADH4 genes.

**Fig. 1:**
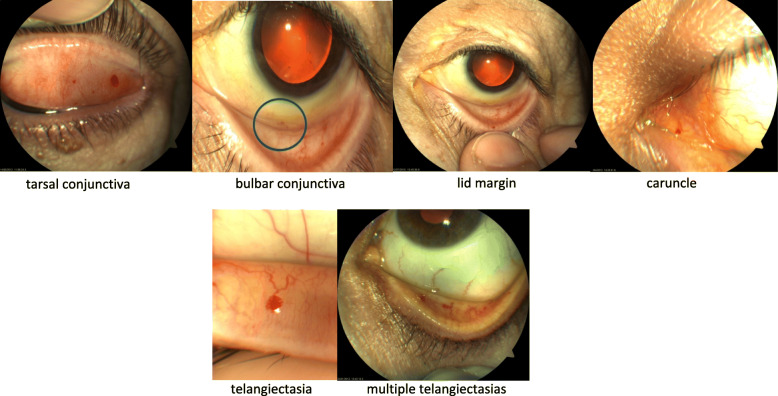
Location of ocular telangiectasia in patients with HHT

### Genotyping procedure (Fig. 1)

All patients were genotyped searching for mutations in ACVRL1/ALK1, ENG and MADH4 genes; if all three genotyped were negative, multiplex ligation-dependent probe amplification (MLPa) techniques were performed to rule out deletions / insertions in ACVRL1/ALK1 and ENG. Results of all genetic tests were compared with the international database of HHT mutations [18] to check whether they were mutations already described or they were new.

### Ophthalmologic examination

All patients were studied in the Ophthalmology service. In the ophthalmological examination, the best corrected visual acuity, the palpebral physical examination and the anterior ocular segment have been assessed by means of a slit lamp, including upper eyelid eversion. The ocular tension was measured by contact tonometry and the fundus was explored under mydriasis by indirect ophthalmoscopy. In case of injuries, these were photographed.

### Other tests

All patients underwent physical examination, family tree scrutiny and search for vascular malformations by cerebral MRI angiography, conventional and contrast (bubbles) echocardiography, conventional abdominal ultrasound, abdominal Doppler to study hepatic vascularization and capillaroscopy evaluating megacapillaries and microhemorrhages in the nail bed of the 4th and 3th fingers of the right hand. All patients were studied in the Otolaryngology service. All patients also received the Quality of Life Scale (Euroqol 5D) and exclusively for women of childbearing age, a lumbar MRI angiography was also performed.

Other explorations could be carried out depending of the results of the previous tests, including thoracic CT with or without intravenous contrast, CT angiography, abdominal CT and pulmonary angiography.

### Statistical analysis

For the description of the main qualitative variables, proportions were used. Quantitative variables were described by mean and standard deviation (SD). To quantify the degree of association between the variables, raw and age- and sex-adjusted odds ratios (OR) with their 95% confidence interval (95% CI) were estimated via logistic regression. In all the statistical tests used, a value of *p* < 0.05 was considered statistically significant. Stata 14 / SE package was used in the analyses.

## Results

### Characteristics of patients

Table 1 displays demographic characteristics and distribution of ocular involvement. Out of 206 patients with confirmed HHT, 105 (51%) had ocular telangiectasias on the examination performed by ophthalmologists. Patients with ocular involvement were older on average (50.3 ± 13.5 vs. 44.7 ± 15.7, *p* = 0.006). Eye lesions did not affect visual acuity or intraocular pressure. Fifty-five patients had both eyes affected; most ocular telangiectasias were located in the tarsal conjunctiva and fewer were located in bulbar conjunctiva, lid margin and caruncle; no lesions were found in retina. Figure 1 shows pictures with different locations of ocular telangiectasias. Regarding the exploration with slit lamp and ophthalmoscopy, we found no differences between patients with and without ocular involvement. Eye involvement was not associated with the accomplished criteria of Curaçao (Table 2). Regarding the association between the patient’s personal history and ocular involvement (supplementary Table 1) we observe that there are no statistically significant differences between patients with arterial hypertension or diabetes mellitus and ocular involvement. Mutations in ACVRL1/ALK1 gene appeared in 118 patients (57%) and in Endoglin gene in 77 patients (37%). Eye involvement was twice as frequent in patients carrying a mutation in the Endoglin gene than in patients with mutation on the ACVRL1/ALK1 gene (Odds ratio [OR] = 2.10, 95% CI: 1.12–3.95) (Table 3).

**Table 1:** Demographic data and eye involvement of patients with HHT

	Eye involvement	No eye involvement	***p*** value
**Sex**			
**Men**	48 (49.5%)	49 (50.5%)	0.69
**Women**	57 (52.3%)	52 (47.7%)	
**Age: mean ± sd**	50.3 ± 13.5	44.7 ± 15.7	0.006
**< 19 years**	1 (11.1%)	8 (88.9%)	0.05
**20–39 years**	24 (45.35)	29 (54.7%)	
**40–59 years**	53 (55.2%)	43 (44.8%)	
**60–79 years**	27 (56.3%)	21 (43.7%)	
**Visual acuity: mean ± sd**			
**Right eye**	0.90 ± 0.17	0.93 ± 0.18	0.26
**Left eye**	0.88 ± 0.22	0.94 ± 0.21	0.11
**Intraocular pressure: mean ± sd**			
**Right eye**	15.40 ± 2.15	15.80 ± 2.17	0.32
**Left eye**	15.21 ± 2.05	15.66 ± 2.03	0.24
**Laterality of ocular telangiectasias**			
**Both eyes**	55		
**Right eye**	34		
**Left eye**	15		
**Conjunctiva location of telangiectasias: patients (no. of eyes)**			
**Tarsal**	97 (150)		
**Bulbar**	2 (2)		
**Border**	6 (7)		
**Caruncle**	1 (1)		
**Eyelid location of telangiectasias: patients (no. of eyes)**			
**Upper and lower**	18 (30)		
**Upper**	32 (42)		
**Lower**	23 (32)

**Table 2:** Eye involvement association with diagnostic criteria of Curaçao

Curaçao criteria		Eye involvement	No eye involvement
**Telangiectasias**	**No**	0 (0%)	0 (0%)
	**Yes**	105 (100%)	101 (100%)
**Epistaxis**	**No**	1 (0.95%)	3 (2.97%)
	**Yes**	104 (99.05%)	98 (97.03%)
**Visceral lesions**	**No**	22 (20.95%)	27 (26.63%)
	**Yes**	83 (79.05%)	74 (73.27%)
**First degree relative affected**	**No**	1 (0.9%)	1 (1.0%)
	**Yes**	104 (99.1%)	100 (99.0%)
**N ° criteria**	**1**	0 (0%)	0 (0%)
	**2**	0 (0%)	1 (1.0%)
	**3**	24 (22.9%)	29 (28.7%)
	**4**	81 (77.1%)	71 (70.3%)

**Table 3:** Eye involvement and genetic mutations

Gene	Eye involvement (***n*** = 105)	No eye involvement (***n*** = 101)	OR (95% CI)	*p*
**ACVRL1/ALK1**	52 (49.5%)	66 (65.3%)	1 (reference)	–
**ENG**	48 (45.7%)	29 (28.7%)	2.10 (1.12–3.95)	0.01
**MADH4**	1 (1.0%)	0 (0%)	–	–
**No mutation founded**	4 (3.8%)	6 (5.9%)	0.85 (0.17–3.79)	0.80

ACVRL1: Activin A receptor like kinase type 1. ENG: Endoglin. MADH4 (Mother against decapentaplegic homolog 4). OR: Odds ratio. CI: Confidence interval

The more frequent locations for telangiectasias -apart from conjunctiva- were nose (192, 93%), lips (166, 81%), mouth (156, 76%) and fingers (128, 62%). Ocular telangiectasias were associated with telangiectasias in fingers (age and sex adjusted OR = 2.25, 95% CI: 1.21–4.17, *p* = 0.01), but not with telangiectasias in other locations as nasal, labial, oral or trunk (Table 4). As with the association of ocular involvement and the presence of telangiectasias in the fingers, ocular lesions in HHT have a significant association with capillaroscopy, multiplying by 2.26 the possibility of presenting a positive result (OR = 2.26; IC95% [1.20–4.27] *p* = 0.004). This probability decreases if we adjust for age and sex (OR = 1.99; IC95% [1.04–3.81]) but remains significant (Supplementary Table 4).

**Table 4:** Association of ocular telangiectasias with telangiectasias in other locations

Telangiectasia location		Eye involvement (yes/total)	OR (95% CI)	*p*	OR^a^ (95% CI)	*p*
**Facial**	**No**	41/96	1 (Reference)		1 (Reference)	
	**Yes**	64/110	1.87 (1.07–3.25)	0.03	1.45 (0.78–2.69)	0.24
**Nasal**	**No**	5/14	1 (Reference)		1 (Reference)	
	**Yes**	100/192	1.96 (0.63–6.05)	0.24	1.45 (0.46–4.64)	0.53
**Lip**	**No**	15/40	1 (Reference)		1 (Reference)	
	**Yes**	90/166	1.97 (0.97–4.01)	0.06	1.67 (0.80–3.47)	0.17
**Oral**	**No**	22/50	1 (Reference)		1 (Reference)	
	**Yes**	83/156	1.45 (0.76–2.75)	0.26	1.07 (0.53–2.14)	0.85
**Fingers**	**No**	28/78	1 (Reference)		1 (Reference)	
	**Yes**	77/128	2.70 (1.51–4.83)	0.001	2.25 (1.21–4.17)	0.01
**Trunk**	**No**	93/178	1 (Reference)		1 (Reference)	
	**Yes**	12/28	0.69 (0.31–1.53)	0.36	0.67 (0.30–1.53)	0.35
**Arms**	**No**	101/202	1 (Reference)		1 (Reference)	
	**Yes**	4/4	–		–	

*OR* Odds ratio, *CI* Confidence interval, *OR*^a^ Odds ratio adjusted for age at onset and sex

In relation to the association of ocular involvement with bleeding in locations other than epistaxis, significant association has been found only with bleeding from lesions at the mouth (age and sex adjusted OR = 2.16; 95% CI: 1.18–3.97, *p* = 0.01) and at the skin of the nose (age and sex adjusted OR = 2.90, 95% CI 1.60–5.26 *p* = 0.001), but not with bleeding in skin, digestive tract or central nervous system (Supplementary Table 2). The relation between eye involvement and epistaxis / nasal telangiectasias is displayed in Supplementary Table 3. Eye telangiectasias were associated with grade II intensity epistaxis (age and sex adjusted OR = 2.03, 95% CI: 1.04–3.97, *p* = 0.04) and with nasal telangiectasias grade (p for trend = 0.001); nasal telangiectasias grade III (branched telangiectasias) (age and sex adjusted OR = 2.52, 95% CI: 1.00–6.34, *p* = 0.05) and grade IVa (isolated vascular malformations) (age and sex OR = 3.84, 95% CI: 1.20–12.3, *p* = 0.02) displayed significant associations with ocular involvement. Finally, Supplementary Table 4 shows the association between pulmonary involvement and ocular telangiectasias. Pulmonary lesions were associated with ocular telangiectasias either when studied by bubble contrast echocardiography (p for trend = 0.03, age and sex adjusted OR for grade 4 lesions = 3.92, 95% CI: 1.25–12.3, p = 0.02), computerized tomography angiography (age and sex adjusted OR = 1.99, 95% CI: 0.98–4.02, *p* = 0.06) or pulmonary angiography (age and sex adjusted OR = 20, 95% CI: 2.42–165.5, *p* = 0.005), although the last result was based in just 25 explorations.

## Discussion

According to our results, ocular involvement in Spanish HHT patients is more frequent in people with mutations in the endoglin gene, which is responsible for HHT subtype 1 (HHT1), than in patients with mutations in the ACVRL1/ALK1 gene, responsible for HHT subtype 2 (HHT2). HHT1 has been associated with higher frequency of arteriovenous malformations located in lungs or in brain, while patients with HHT2 are more frequently affected of telangiectasias in the liver [19]. Coherently, we have found that ocular manifestations of HHT are associated with lung involvement as measured via bubble contrast echocardiography or computed tomography-angiography, thus ocular telangiectasias could serve as a non-invasive marker of HHT manifestations in the respiratory tract. Other signs of HHT severity, such as oral bleeding, telangiectasias in the nostril, grade of nasal telangiectasias and epistaxis intensity are also associated with ocular telangiectasias, according to our results. In this regard Bergh et al., found that HHT1 subtype patients suffer of larger congenital lesions and, thus, they considered HHT 1 as a more severe phenotype [20]. On the other hand, Lesca et al. [21] studied the association of telangiectasias in lips, fingers, tongue, and nose, finding a high penetration in HHT1 with a greater number of telangiectasias in this subtype. These findings are in agreement with our results. In this same way, presumably there is an association between abnormal capillaroscopy and ocular involvement in our study because capillaroscopy is more altered in HHT1 [1].

In the only previous study analysing the relationship between conjunctival telangiectasias and HHT subtype [22], the percentages of patients with conjunctival involvement were 13.4% for HHT1 subtype and 14.6% for HHT2 subtype, which disagrees with our results. However, the ocular exploration in that study was not performed by ophthalmologists, which makes its prevalence values of ocular disease not completely comparable to ours. Other studies that have found mucocutaneous telangiectasias were unrelated to genetic mutations, either did not specify the telangiectasias location or studied them only in typical mucocutaneous locations that are considered in the Curaçao criteria, i.e., they did not consider ocular involvement [4, 19–21, 23].

Besides that, in our study, the type of ocular lesion presented by patients with HHT are mainly telangiectasias or conjunctival vascular malformations with low rate of symptoms and only 1.12% prevalence of retinal involvement. However, we have not found any patient with retinal lesion. One of the reasons for our low retinal prevalence may be due to the fact that in the ophthalmology unit the new optical coherence tomography modality, angiographic, was not available, which could have allowed detailed study of retinal and even choroid vascularization, without the needing of using contrast. Therefore, subtle vascular retinal abnormalities may have been overlooked in the subjects who did not have fundoscopic abnormalities and, consequently, were not examined by fluorescein angiography (FA) (which is an invasive test, relegated to specific cases). However, relatively well preserved visual acuity in our cohort of participants could support that there were no retinal lesions or, as a recent article with OCT-Angiography and wide-field angiography data from 24 eyes has revealed, that intraocular lesions in patients with HHT predominantly affect peripheral vasculature, while retinal vascular diseases, such as diabetes and age-related macular degeneration, typically affect the more metabolically active central vasculature of the retina [16]. Indirect ophthalmoscopy could have not been accurate enough to rule out the presence of discrete peripheral retinal telangiectasias, which can only be easily seen by UWF-FA. However, UWF-FA is an invasive test not indicated in our patients and OCT-Angiography was not available on the dates of our study. Further research with this technique in a greater number of subjects with HHT would be necessary to more accurately assess the presence of retinal lesions. Further research with this technique in a greater number of subjects with HHT would be necessary to more accurately assess the presence of retinal lesions. In addition, it is noteworthy that telangiectasias seem to have predilection for certain organs, the conjunctiva being the most affected location in the eye instead of other more vascularized locations such as the choroid.

This study has some strengths. Firstly, all patients included in this study have a confirmed genetic diagnosis and have been followed by professionals specialized in the manifestations of the disease and its management. When Vase and Vase [17], in 1979, and Brant et al. [13] in 1989 carried out their studies on the prevalence of ocular involvement in HHT, the diagnostic criteria of Curaçao had not been established and the techniques of current genetic diagnosis were not available, so the number of patients diagnosed with HHT in their studies does not fit the current diagnostic criteria of the disease. Secondly, the HHT Unit of Hospital de Sierrallana is a reference centre for Spanish patients with HHT and the ophthalmological examination is included in the routine protocol of the HHT Unit; therefore, patients included in our study have not been selected because of suspicion of ocular involvement, which rules out selection bias. In addition, participation of ophthalmologists in one previous study in 2007 on ocular involvement [14] was carried out through questionnaires that had been submitted to the research centre.

In our series of 206 patients there are 105 patients with ocular lesions, so the prevalence of ocular involvement in our HHT population is 51%. Three studies that analysed the clinical characteristics in populations of high prevalence of HHT provided much lower prevalence of conjunctival involvement, with values of 1% [24], 14% [22] and 16% [2]. However, these studies do not include the participation of ophthalmologists. On the other hand, five comparable prevalence studies have been published in the literature [13–17] with case series of 20, 75, 8, 18 and 43, patients respectively, who have been examined by ophthalmologists, so that our study represents the one with the highest number of cases published to date. These studies estimate the prevalence of ocular involvement at 43% [17], 35% [13] and 38% [14] for conjunctival lesions and 2, 10 and 0% for retinal lesions. Notice there is a big difference between the prevalence values of ocular involvement between studies published in the literature. This highlights the importance of a good examination by an ophthalmologist to confirm or rule out the existence of ocular lesions related to HHT. In addition, according to our data, patients with ocular telangiectasias were older; although, the natural history of vascular malformations in HHT patients is not well understood, this result suggests that ocular telangiectasias may appear in later stages of HHT [16].

## Conclusions

Half of the patients with HHT in our study have ocular involvement. The type of ocular lesion that our patients present are mainly conjunctival telangiectasias with low rate of symptoms and are associated with HHT 1 subtype and several signs of disease severity. In addition, as the probability of eye involvement subject to mutation in ENG is higher than that in ALK1 (62.34% vs. 44.07%), we suggest starting the genetic sequencing with ENG if mutated, or ALK1 otherwise. Further studies are needed to confirm these results, which suggest ocular telangiectasias could be a non-invasive marker of severity in HHT.

## Supplementary information

**Additional file 1: Supplementary Table 1.** Association of ocular telangiectasias with personal history. **Supplementary Table 2.** Association of ocular involvement with bleeding in other locations. **Supplementary Table 3.** Association of ocular involvement with epistaxis characteristics. **Supplementary Table 4**. Association of ocular telangiectasias with lung involvement.

## Data Availability

Permission to use the study database will be granted to researchers outside the study group after revision and approval of each request by the Steering Committee.

## Abbreviations

ACVRL1: Activin A receptor like kinase type 1
ENG: Endoglin MADH4 (Mother against decapentaplegic homolog 4)
OR: Odds ratio
CI: Confidence interval
