# Supplementary material for: Ocular lesions in hereditary hemorrhagic telangiectasia: genetics and clinical characteristics
Source: Orphanet J Rare Dis. 2020 Jun 29;15:168. doi: 10.1186/s13023-020-01433-5 (PMC7322834; doi:10.1186/s13023-020-01433-5)
Supplement: Supplementary file 1 — Additional file 1: Supplementary Table 1. Association of ocular telangiectasias with personal history. Supplementary Table 2. Association of ocular involvement with bleeding in other locations. Supplementary Table 3. Association of ocular involvement with epistaxis characteristics. Supplementary Table 4. Association of ocular telangiectasias with lung involvement. [file 13023_2020_1433_MOESM1_ESM.docx]

**Supplementary table 1. Association of ocular telangiectasias with personal history**

|  | **Eye involvement (yes/total)** | **OR (95% CI)** | **p** | **OR^1^ (95% CI)** | **p** |
| --- | --- | --- | --- | --- | --- |
| **Respiratory** |  |  |  |  |  |
| **NO** | 75/151 | 1(Reference) |  | 1(Reference) |  |
| **YES** | 30/55 | 1.22 (0.65-2.26) | 0.536 | 1.21 (0.64-2.28) | 0.561 |
| **Neurological** |  |  |  |  |  |
| **NO** | 175/254 | 1(Reference) |  | 1(Reference) |  |
| **YES** | 30/52 | 1.44 (0.76-2.71) | 0.263 | 1.51 (0.79-2.89) | 0.215 |
| **Gastrointestinal** |  |  |  |  |  |
| **NO** | 68/144 | 1(Reference) |  | 1(Reference) |  |
| **YES** | 37/62 | 1.65 (0.90-3.03) | 0.102 | 1.22 (0.63-2.36) | 0.562 |
| **Liverworts** |  |  |  |  |  |
| **NO** | 99/194 | 1(Reference) |  | 1(Reference) |  |
| **YES** | 6/12 | 0.96 (0.30-3.08) | 0.945 | 0.74 (0.22-2.45) | 0.620 |
| **Cardiac** |  |  |  |  |  |
| **NO** | 88/180 | 1(Reference) |  | 1(Reference) |  |
| **YES** | 17/26 | 1.97 (0.84-4.66) | 0.121 | 1.73 (0.71-4.23) | 0.231 |
| **Menopause** |  |  |  |  |  |
| **NO** | 78/156 | 1(Reference) |  | 1(Reference) |  |
| **YES** | 40/63 | 1.17 (0.62-2.22) | 0.622 | 0.53 (0.21-1.30) | 0.166 |
| **HBP** |  |  |  |  |  |
| **NO** | 84/171 | 1(Reference) |  | 1(Reference) |  |
| **YES** | 21/35 | 1.55 (0.74-3.26) | 0.243 | 0.98 (0.42-2.30) | 0.968 |
| **DM** |  |  |  |  |  |
| **NO** | 99/197 | 1(Reference) |  | 1(Reference) |  |
| **YES** | 6/9 | 1.98 (0.48-8.14) | 0.344 | 1.45 (0.34-6.26) | 0.620 |
| **PET/PVT** |  |  |  |  |  |
| **NO** | 100/196 | 1(Reference) |  | 1(Reference) |  |
| **YES** | 5/10 | 0.96 (0.27-3.42) | 0.950 | 0.83 (0.23-3.01) | 0.772 |
| **ACVA** |  |  |  |  |  |
| **NO** | 91/185 | 1(Reference) |  | 1(Reference) |  |
| **YES** | 14/21 | 2.07 (0.80-5.35) | 0.135 | 2.08 (0.78-5.54) | 0.141 |
| **Ischemic Heart Disease** |  |  |  |  |  |
| **NO** | 99/197 | 1(Reference) |  | 1(Reference) |  |
| **YES** | 6/9 | 1.98 (0.48-8.14) | 0.344 | 1.60 (0.37-6.90) | 0.528 |
| **Oestrogen treatment** |  |  |  |  |  |
| **NO** | 95/188 | 1(Reference) |  | 1(Reference) |  |
| **YES** | 10/18 | 1.22 (0.46-3.24) | 0.684 | 1.40 (0.51-3.89) | 0.516 |
| **Tobacco** |  |  |  |  |  |
| **NO** | 81/162 | 1(Reference) |  | 1(Reference) |  |
| **YES** | 24/44 | 1.20 (0.61-2.34) | 0.593 | 1.29 (0.65-2.58) | 0.470 |
| **Medical allergies** |  |  |  |  |  |
| **NO** | 75/156 | 1(Reference) |  | 1(Reference) |  |
| **YES** | 30/50 | 1.62 (0.85-3.09) | 0.144 | 1.55 (0.80-3.01) | 0.197 |
| **Other background** |  |  |  |  |  |
| **NO** | 12/28 | 1(Reference) |  | 1(Reference) |  |
| **YES** | 93/178 | 1.46 (0.65-3.26) | 0.358 | 1.19 (0.52-2.74) | 0.687 |

HBP: high blood pressure. DM: diabetes mellitus. PET/PVT: pulmonary thromboembolism/deep vein thrombosis. ACVA: stroke

**OR: Odds ratio. CI: Confidence interval. OR^1^: Odds ratio adjusted for age onset and sex**

**Supplementary table 2. Association of ocular involvement with bleeding in other locations**

| **Bleeding location** |  | **Eye involvement (yes/total)** | **OR (95% CI)** | **p** | **OR^1^ (95% CI)** | **p** |
| --- | --- | --- | --- | --- | --- | --- |
| **Oral** | **No** | 59/135 | 1 (Reference) |  | 1 (Reference) |  |
|  | **Yes** | 46/71 | 2.37 (1.31-4.29) | 0.004 | 2.16 (1.18-3.97) | 0.01 |
| **Cutaneous** | **No** | 79/165 | 1 (Reference) |  | 1 (Reference) |  |
|  | **Yes** | 26/41 | 1.89 (0.93-3.82) | 0.08 | 1.60 (0.77-3.31) | 0.21 |
| **Digestive** | **No** | 90/179 | 1 (Reference) |  | 1 (Reference) |  |
|  | **Yes** | 15/27 | 1.24 (0.55-2.79) | 0.61 | 0.92 (0.39-2.17) | 0.84 |
| **High digestive** | **No** | 89/179 | 1 (Reference) |  | 1 (Reference) |  |
|  | **Yes** | 16/27 | 1.47 (0.65-3.35) | 0.36 | 1.08 (0.45-2.59) | 0.86 |
| **Low digestive** | **No** | 104/203 | 1 (Reference) |  | 1 (Reference) |  |
|  | **Yes** | 1/3 | 0.48 (0.04-5.33) | 0.55 | 0.43 (0.04-4.95) | 0.50 |
| **Central Nervous System** | **No** | 103/202 | 1 (Reference) |  | 1 (Reference) |  |
|  | **Yes** | 2/4 | 0.96 (0.13-6.96) | 0.97 | 0.94 (0.13-6.92) | 0.95 |
| **Skin of the nose** | **No** | 43/113 | 1 (reference) | - | 1 (reference) | - |
|  | **Yes** | 62/93 | 3.26 (1.83-5.78) | 0.001 | 2.90 (1.60-5.26) | 0.001 |

**OR: Odds ratio. CI: Confidence interval. OR^1^: Odds ratio adjusted for age at onset and sex**

**Supplementary table 3. Association of ocular involvement with epistaxis characteristics**

|  | **Eye involvement (yes/total)** | **OR (95% CI)** | **p** | **OR^1^ (95% CI)** | **p** |
| --- | --- | --- | --- | --- | --- |
| **Severity** |  |  |  |  |  |
| **None** | 1/3 | 0.60 (0.05-6.78) | 0.68 | 0.45 (0.04-5.25) | 0.52 |
| **Mild** | 47/103 | 1(ref.) | - | 1(ref.) | - |
| **Moderate** | 34/62 | 1.45 (0.77-2.72) | 0.25 | 1.18 (0.61-2.28) | 0.63 |
| **Severe** | 23/38 | 1.83 (0.86-3.90) | 0.12 | 1.43 (0.64-3.17) | 0.38 |
| **Frequency (Bergler et al, 2002)** |  |  |  |  |  |
| **Grade 1 – less than once per week** | 20/51 | 1 (Reference) | - | 1 (Reference) | - |
| **Grade 2 – several times per week** | 37/69 | 1.79 (0.86-3.74) | 0.12 | 1.59 (0.75-3.37) | 0.23 |
| **Grade 3 – more than once per day** | 48/86 | 1.96 (0.97-3.96) | 0.06 | 1.60 (0.77-3.34) | 0.21 |
| **Intensity (Bergler et al, 2002)** |  |  |  |  |  |
| **Grade I: slight stains on handkerchief** | 26/67 | 1 (Reference) | - | 1 (Reference) | - |
| **Grade II: soaked handkerchief** | 53/91 | 2.20 (1.15-4.19) | 0.02 | 2.03 (1.04-3.97) | 0.04 |
| **Grade III: bowl or similar utensil necessary** | 26/48 | 1.86 (0.88-3.95) | 0.10 | 1.34 (0.59-3.03) | 0.48 |
| **Seasonal Variations** |  |  |  |  |  |
| **No** | 62/121 | 1 (Reference) | - | 1 (Reference) | - |
| **Yes** | 43/85 | 0.97 (0.56-1.70) | 0.93 | 0.99 (0.56-1.75) | 0.98 |
| **Progression with age** |  |  |  |  |  |
| **No** | 1/4 | 1 (Reference) |  | 1 (Reference) |  |
| **Yes** | 104/202 | 3.18 (0.33-31.1) | 0.32 | 3.48 (0.35-34.6) | 0.29 |
| **Grade of nasal telangiectasias** | |  |  |  |  |
| **0:** | 6/15 | 1.20 (0.36-4.03) | 0.77 | 1.02 (0.30-3.53) | 0.97 |
| **I: isolated punctate telangiectasias** | 15/42 | 1(ref.) | - | 1(ref.) | - |
| **II: multiple punctate telangiectasias** | 33/72 | 1.52 (0.70-3.33) | 0.29 | 1.19 (0.52-2.72) | 0.68 |
| **III: branched telangiectasias** | 25/40 | 3.00 (1.22-7.37) | 0.02 | 2.52 (1.00-6.34) | 0.05 |
| **IVa- isolated vascular malformations** | 17/23 | 5.10 (1.66-15.7) | 0.005 | 3.84 (1.20-12.3) | 0.02 |
| **IVb-multiple vascular malformations** | 9/14 | 3.24 (0.92-11.45) | 0.07 | 2.43 (0.66-8.94) | 0.18 |
| **No. tampons** |  |  |  |  |  |
| **0** | 97/192 | 1 (Reference) | - | 1 (Reference) | - |
| **1** | 1/3 | 0.49 (0.04-5.49) | 0.56 | 0.36 (0.03-4.15) | 0.42 |
| **2** | 0/1 |  |  |  |  |
| **Transfusions** |  |  |  |  |  |
| **No** | 80/166 | 1 (Reference) | - | 1 (Reference) | - |
| **Yes** | 16/28 | 1.88 (0.97-3.67) | 0.38 | 1.33 (0.63-2.78) | 0.45 |
| **Hospital admissions** |  |  |  |  |  |
| **No** | 69/147 | 1 (Reference) | - | 1 (Reference) | - |
| **Yes** | 30/48 | 1.43 (0.97-3.67) | 0.06 | 1.06 (0.45-2.53) | 0.89 |

**OR: Odds ratio. CI: Confidence interval. OR^1^: Odds ratio adjusted for age at onset and sex**

**Supplementary table 4. Association of ocular telangiectasias with lung involvement**

|  | **Eye involvement (yes/total)** | **OR (95% CI)** | **p** | **OR^1^ (95% CI)** | **p** |  |  |
| --- | --- | --- | --- | --- | --- | --- | --- |
| **Thorax X-ray** |  |  |  |  |  |  |  |
| **Negative** | 86/172 | 1(Reference) |  | 1(Reference) |  |  |  |
| **Pathological** | 13/23 | 1.30 (0.54-3.12) | 0.56 | 1.43 (0.57-3.54) | 0.45 |  |  |
| **Standing oximetry** |  |  |  |  |  |  |  |
| **Normal** | 88/171 | 1(Reference) |  | 1(Reference) |  |  |  |
| **Low** | 9/16 | 1.21 (0.43-3.40) | 0.71 | 1.38 (0.46-4.19) | 0.57 |  |  |
| **Bubble Contrast Echocardiography** | |  |  |  |  |  |  |
| **Grade 0** | 22/53 | 1(Reference) |  | 1(Reference) |  |  |  |
| **Grade 1** | 36/75 | 1.30 (0.64-2.64) | 0.47 | 1.27 (0.61-2.64) | 0.52 |  |  |
| **Grade 2** | 21/38 | 1.74 (0.75-4.04) | 0.20 | 1.84 (0.78-4.36) | 0.17 |  |  |
| **Grade 3** | 8/16 | 1.41 (0.46-4.33) | 0.55 | 1.73 (0.53-5.61) | 0.36 |  |  |
| **Grade 4** | 14/20 | 3.29 (1.09-9.89) | 0.03 | 3.92 (1.25-12.3) | 0.02 |  |  |
| **Computed tomography angiography** | |  |  |  |  |  |  |
| **Normal** | 59/123 | 1(Reference) |  | 1(Reference) |  |  |  |
| **Pathological** | 31/48 | 1.98 (0.99-3.94) | 0.05 | 1.99 (0.98-4.02) | 0.06 |  |  |
| **Pulmonary angiography** | |  |  |  |  |  |  |
| **Normal** | 2/7 | 1(Reference) |  | 1(Reference) |  |  |  |
| **Pathological** | 16/18 | 13.3 (1.71-103.8) | 0.01 | 20 (2.42-165.5) | 0.005 |  |  |
| **Capillaroscopy** |  |  |  |  |  |  |  |
| **Negative** | 35/76 | 1(Referencia) |  | 1(Referencia) |  |  |  |
| **Positive** | 56/85 | 2,26 (1,20-4,27) | 0,01 | 1,99 (1,04-3,81) | 0,039 |  |  |

**OR: Odds ratio. CI: Confidence interval. OR^1^: Odds ratio adjusted for age onset and sex**
